# Diverse Genetic Background of Multidrug-Resistant *Pseudomonas aeruginosa* from Mainland China, and Emergence of an Extensively Drug-Resistant ST292 Clone in Kunming

**DOI:** 10.1038/srep26522

**Published:** 2016-05-20

**Authors:** Xin Fan, Yue Wu, Meng Xiao, Zhi-Peng Xu, Timothy Kudinha, Alda Bazaj, Fanrong Kong, Ying-Chun Xu

**Affiliations:** 1 Department of Clinical Laboratory, Peking Union Medical College Hospital, Chinese Academy of Medical Sciences, Beijing, China; 2 Graduate School, Peking Union Medical College, Chinese Academy of Medical Sciences, Beijing, China; 3 Charles Sturt University, Leeds Parade, Orange, New South Wales, Australia; 4 Centre for Infectious Diseases and Microbiology Laboratory Services, ICPMR – Pathology West, University of Sydney, Westmead Hospital, Darcy Road, Westmead, New South Wales, NSW 2145, Australia

## Abstract

For a better understanding of the multidrug resistant *Pseudomonas aeruginosa* (MDR-PA) epidemiology in mainland China, a nationwide surveillance network of 27 tertiary hospitals was established. Non-duplicate MDR-PA isolates from 254 cases of nosocomial infections, were collected during the period August 2011 to July 2012. Minimum inhibitory concentrations (MICs) of nine antimicrobial agents were determined by broth micro-dilution method according to the CLSI guidelines [M7-A10]. Genotyping analysis was performed by multilocus sequence typing (MLST) and pulsed-field gel electrophoresis (PFGE). The presence of acquired carbapenemases was also determined by molecular approaches for 233 carbapenem-resistant isolates. Carbapenemase genes were detected in 19 (8.2%) isolates, with 13 of these isolates encoding IMP-type enzymes, five with VIM-2, and one with KPC-2. MLST analysis revealed significant genetic diversity among the MDR-PA isolates studied, and 91 STs (including 17 novel STs) were identified. However, a long-term outbreak of an emerging extensively drug-resistant (XDR) ST292/PFGE genotype A clone was detected in a hospital from Southwest China. This study has demonstrated that MDR-PA in mainland China have evolved from diverse genetic backgrounds. Evidence of clonal dissemination of the organism and nosocomial outbreaks in some regions, suggest a need to strengthen existing infection control measures.

*Pseudomonas aeruginosa* is one of the most common nosocomial pathogens, especially among patients in intensive care units, burn centres, and cystic fibrosis centres. Given its intrinsic resistance to a large variety of antimicrobials^1^,^2^, the antimicrobial drug choices for infections caused by this pathogen are more limited than those for other Gram negative bacilli. Furthermore, *P. aeruginosa* can acquire resistance determinants by horizontal transfer of mobile genetic elements from other bacteria. Infections caused by multidrug resistant *P. aeruginosa* (MDR-PA) or extensively drug-resistant (XDR-PA) and pan-drug-resistant *P. aeruginosa* (PDR-PA)^3^, are extremely difficult to treat and pose a great challenge to both physicians and patients.

Carbapenems are important antimicrobial agents for the treatment of *P. aeruginosa* infections. However, increasing resistance to these compounds by *P. aeruginosa* has restricted their use in many geographical areas^1^,^2^,^4^,^5^. Although carbapenem resistance in *P. aeruginosa* may occur through different mechanisms, the acquired carbapenemases are of the utmost concern, as they are characterized by a very wide hydrolytic spectrum and affect almost all β-lactams. The two major groups of carbapenemases commonly found in *P. aeruginosa*, namely IMP and VIM, are Ambler Class B metallo-β lactamases (MBLs). Ambler class A carbapenemases, such as *Klebsiella pneumoniae* carbapenemase (KPC), are more frequently reported in *Enterobacteriaceae*, although they have started to be detected in *P. aeruginosa* isolates as well^6^. Given the increasing prevalence of carbapenemases and the limited information available about their prevalence in mainland China, we decided to investigate the frequency of different types of carbapenemases among MDR-PA isolates in this geographic area (Fig. 1).

Although several studies have shown that the *P. aeruginosa* population structure is typically non-clonal^7^,^8^, several other studies have described the presence of MBL-producing clones in hospitals^9^,^10^. For a comprehensive understanding of the MDR-PA problem in China, we have established a nationwide surveillance network of 27 tertiary hospitals from diverse geographical areas (Fig. 1) to monitor this pathogen, including antimicrobial susceptibilities, molecular epidemiology, clonal structure and prevalence rate of carbapenemase in nosocomial MDR-PA.

## Results

### General information

A total of 254 non-duplicate MDR-PA isolates were collected from nosocomial infections from 23 of the 27 participating hospitals (four hospitals did not isolate any). The mean age of these 254 patients was 54.6 ± 21.6 years, and 66.1% (168 patients) were male. Among them, 49 patients had skin and soft tissue infection, 44 had bacteraemia, and 42 had urinary tract infections. The main clinical wards in which MDR-PA were isolated included surgical (99 patients, 39.0%) and ICU (68 patients, 26.8%), while the medical ward accounted for 18.9% (48 patients) and all other wards combined accounted for 15.4% (39 patients).

Pus was the most common specimen, accounting for 19.3% of all MDR isolates, followed by blood cultures (17.3%), urine (16.5%), and broncho-alveolar lavage fluid (12.6%). The isolates were also obtained from venous catheters (7.5%), ascitic fluid (5.9%), surgery incision (5.9%), bile (5.5%), hydrothorax (4.7%) and cerebrospinal fluid (3.2%). Tissue biopsy specimens, peritoneal dialysate fluid and bone marrow only yielded one or two isolates each (Table 1).

**Figure 1:**
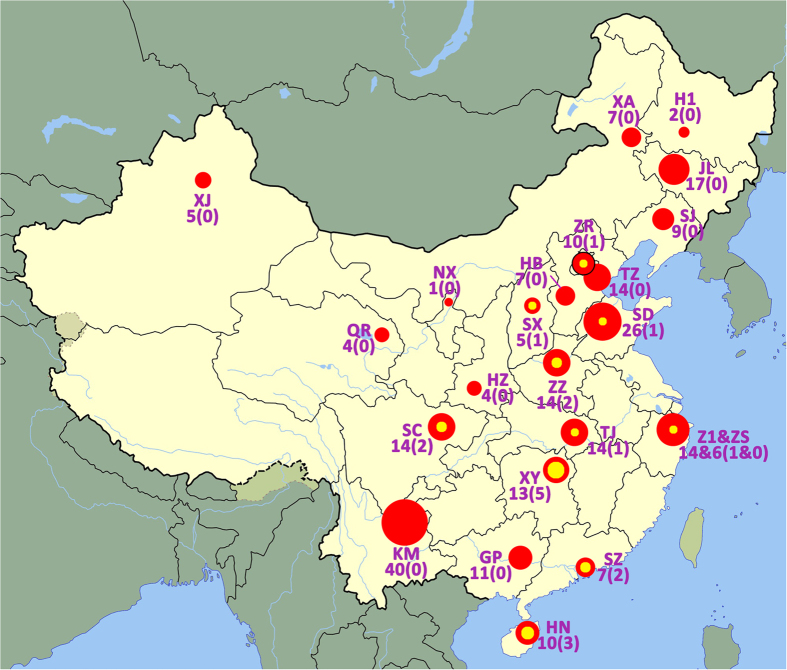
Geographical distribution of multidrug-resistant *Pseudomonas aeruginosa* (MDR-PA) isolates and carbapenemases producers collected in this study. The size of the red cycle and numbers indicate the number of MDR-PA strains; the yellow cycle and the numbers in parentheses indicate the number of carbapenemases producers in different hospitals. The two letters are the abbreviation of the hospitals that participated in this program, and the full hospital name can be found in the Acknowledgements section. Note: the figure was modified from China Blank Map from https://commons.wikimedia.org/wiki/File:China_blank_map.svg.

Among the 254 MDR-PA isolates included in this study, 40 (15.7%) isolates were collected from hospital KM, followed by 26 (10.2%) from hospital SD, 17 (6.7%) from hospital JL. A further 14 isolates (5.5%) each were collected from 5 hospitals (SC, ZZ, TJ, Z1, TZ), followed by 13 (5.1%) from hospital XY. The remaining hospitals contributed on average less than 2.5% of the isolates each (Fig. 1). The ICU (15 isolates), neurosurgery (9 isolates), haemodialysis centre (8 isolates), and urology surgery (5 isolates) were the main wards/departments from which MDR-PA isolates were collected from hospital KM. However, such isolates were also collected in other wards e.g. respiratory and gastroenterology wards.

### Antimicrobial susceptibility

More than 70% of MDR-PA isolates were resistant to piperacillin/tazobactam, cefepime, aztreonam, imipenem, meropenem and ciprofloxacin (Table 2). Colistin was the most active agent (7.9% resistance), followed by amikacin with a resistance rate of 33.5%.

Eighty-six antimicrobial resistance patterns were detected among the 254 MDR-PA isolates. The most common pattern was resistance to piperacillin/tazobactam, cefepime, aztreonam, imipenem, meropenem and ciprofloxacin, but susceptible to ceftazidime, amikacin and colistin (44 isolates, 17.3%). The second most common pattern was resistance to all the agents except colistin (31 isolates, 12.2%). The other antibiotic susceptibility patterns were highly variable, with less than 20 isolates per pattern. The resistance patterns showed no obvious correlation with specimen sources, but with geographical areas. Among 44 isolates sharing the most common pattern, 40 were from KM hospital, and were identified as XDR-PA. Furthermore, among 31 isolates of the second most common pattern, 11 isolates were from the SD hospital.

### Genotyping analysis

Multilocus sequence typing (MLST) analysis revealed significant genetic diversity among MDR-PA, with 91 sequence types (STs) (17 of which were novel) identified among the 254 isolates studied. All the STs belonged to different singletons. The most common ST was ST292 (47 isolates, 18.5%), followed by ST244 (24 isolates, 9.4%), ST277 (18 isolates, 7.1%), ST235 (13 isolates, 5.1%) and ST699 (10 isolates, 3.9%). Seventeen novel STs identified in the present study have been deposited in the MLST database, with assigned numbers of ST1950, ST1956, ST1960 and ST1963 to ST1976 (Supplementary Table S1).

**Figure 2:**
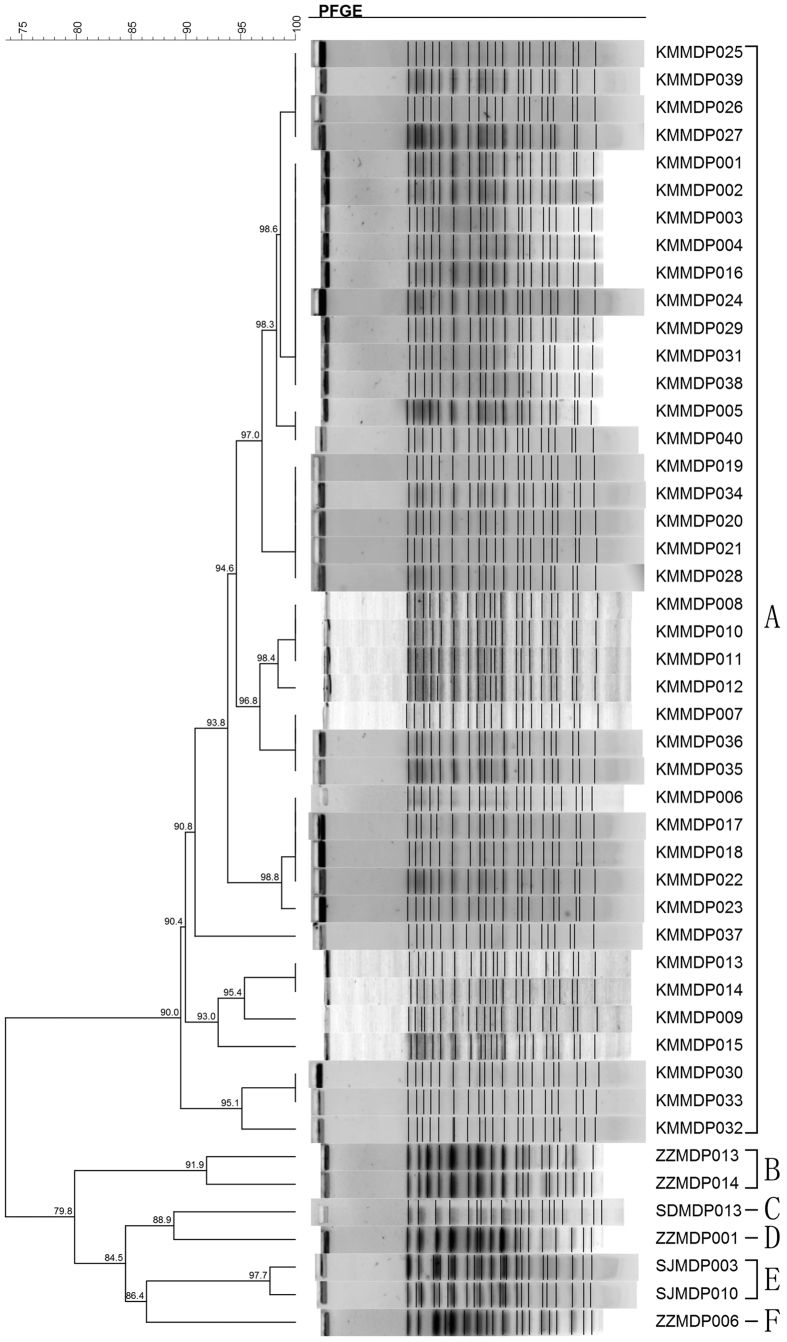
Dendrogram and cluster analysis of 47 ST292 multidrug-resistant *Pseudomonas aeruginosa* isolates detected in the present study by pulsed-field gel electrophoresis (PFGE). The number indicates the coefficient of similarity. For each genotype A–F, they were defined as coefficient of similarity ≥90%.

Pulsed-field gel electrophoresis (PFGE) was performed on all the 47 ST292 (the single most common ST) isolates, and these were discriminated into six different genotypes (A–F) (Fig. 2). A high degree of genetic similarity (≥90%) was observed within the 40 isolates collected from KM hospital and belonged to genotype A. The other 7 isolates from 3 hospitals in different cities, belonged to five genotypes (B–F) (Fig. 2).

### Carbapenemases

Nineteen (8.15% overall) carbapenem-non-susceptible *P. aeruginosa* isolates carried acquired carbapenemase genes, and most of them were isolated from east or south of China (Fig. 1). PCR analysis and sequencing identified 13 isolates encoding IMP-type enzymes (10 IMP-9, two encoding IMP-1 and one encoding IMP-10), five isolates encoding VIM-2, and one isolate encoding KPC-2. The IMP-9-encoding gene was the most common, being detected among 10 isolates from six hospitals (Table 3). Carbapenemase producers were isolated more frequently from urinary tract specimens (8/42, 19.0%) than from other specimens (Table 1). All the 19 carbapenemase producing isolates were resistant or intermediate to β-lactam antimicrobials and β-lactam/β-lactamase inhibitors. Among them, 15 isolates were non-susceptible to ciprofloxacin, whilst 12 and 17 were non-susceptible to amikacin and colistin, respectively.

The MLST ST distribution of the 19 carbapenemase producing isolates was scattered, but three patients hospitalized in the same neurosurgery ward in HN hospital (Fig. 2), had urinary tract infection by IMP-9-producing *P. aeruginosa* each within 45 days, and shared the same resistance pattern and belonged to ST357. Three VIM-2-producing isolates from XY Hospital, but collected from different wards, belonged to different sequence types (STs), and also exhibited different resistance patterns (Table 3).

## Discussion

According to previous studies, clonal dissemination of *P. aeruginosa* is considered to be low^7^,^11^,^12^. In the present study, except for the isolates collected in KM hospital, the MDR-PA isolates exhibited a high genetic diversity and varied resistant patterns. However, it was unusual that all the 40 MDR-PA isolates (40 patients) collected from one hospital (KM) presented the same XDR resistant profiles, belonged to ST292 clone and PFGE genotype A. Clinical records showed that these 40 patients were diagnosed with different infectious diseases and hospitalized in different wards. So we highly suspected that an outbreak of XDR *P. aeruginosa* was present in KM hospital (Yunnan province) during the surveillance period.

There are very limited clinical reports on *P. aeruginosa* ST292 lineage worldwide, with some detailed information on this clone available in a study by Lee *et al.* from Korea^13^. In contrast to the *P. aeruginosa* ST292 isolates in the present study (from KM hospital), the ones in the Korean study were carbapenem-susceptible but had reduced susceptibility to colistin^13^. It is worth noting that the isolates from KM hospital did not produce any carbapenemases. According to previous studies, apart from production of carbapenemases, the major mechanism of carbapenem-resistance in *P. aeruginosa* is the loss of outer membrane porin OprD and over-expression of efflux pump systems MexAB-OprM^16^,^17^. Furthermore, ST292 isolates in the present study had an unusual phenotype, being resistant to cefepime but susceptible to ceftazidime. Previous studies suggested this uncommon phenotype may be due to hyper-expression of the multidrug efflux pump named MexXY-OprM efflux pumps^14^,^15^.

However, possible nosocomial outbreaks of carbapenemase-producing *P. aeruginosa* were actually noted in some other hospitals^9^,^18^,^19^,^20^. In this study, we detected three ST357 MDR-PA isolates, all from three urinary tract infection patients hospitalized in the same neurosurgery ward in HN within 45 days, suggesting possible clonal dissemination. These strains produced IMP-9 and were only susceptible to colistin. ST357 strains have been detected in other geographical regions, but usually produce IMP-7 in Europe^21^,^22^,^23^, and other IMP enzymes like in Japan^24^, which is different from the present study.

Recent studies have revealed significant differences in the prevalence and distribution of carbapenemase-producing *P. aeruginosa* strains from Europe and Asia. In a very recent survey in Europe, performed on 529 carbapenem-non-susceptible *P. aeruginosa*, 20.0% (106 isolates) were positive for MBL, with the VIM-type enzymes^12^ predominating. Only two IMP-type enzymes (IMP-15 and IMP-33) were detected in two strains. However, studies in Japan in 2004 and 2006, detected 2.3% and 2.1% MBL-producers, respectively^24^. A nation-wide survey in mainland China involving 258 *P. aeruginosa* isolates collected from 2006 to 2007 at 28 hospitals, detected MBL in only 22 isolates (8.5%)^11^. Thus comparing to the European data^12^, the carbapenemase prevalence in China and Japan was much lower. In this study, the carbapenemase producing isolates were frequently detected in east and south of China (Fig. 1), regions with larger population densities and considered to be relatively more developed economically than others, and hence probably used drugs more often than other regions.

Previous studies show that the prevalence of MBL producers in Europe kept increasing during the years 2009 to 2011, rising from 12.3% to 30.6%^12^. Based on this background, it is important to adopt and implement continuous surveillance programs for such organisms to assess the effectiveness of current control strategies as well as formulation of new ones. Our study is an important update on the prevalence of carbapenemase producing *P. aeruginosa* in China. The MBL were detected in only 19 isolates (8.15%), and with IMP-9 the most common carbapenemase, which appears to be an important feature of Chinese strains according to previous studies^11^,^25^.

ST235, another recognized international drug-resistant lineage^26^, is more often associated with worldwide outbreaks of nosocomial infections^27^,^28^. In the present study, ST235 was detected in seven hospitals and accounted for 13 isolates (5.1%), but none of them produced carbapenemase. Another potential outbreak case was observed in SD hospital, where ten ST244 *P. aeruginosa* isolates collected from this hospital exhibited a similar resistant profile (Fig. 1). Interestingly, ST244 is a lineage known world-wide to cause outbreaks of nosocomial infections^7^,^29^,^30^.

In conclusion, this study has demonstrated that MDR-PA of nosocomial origin in mainland China are of diverse genetic backgrounds, but suspected nosocomial outbreaks were actually noted in KM hospital, which would suggest strengthening the existing infection control measures for containment of MDR strains.

## Material and Methods

### Ethical approval

This study was approved by the Human Research Ethics Committee of Peking Union Medical College Hospital (Beijing, China) [No. PUMCHBC-C-2-Q01-1], and was carried out strictly in accordance with the approved guidelines. Informed consent was obtained from all subjects.

### Study design

This study was part of a Chinese nationwide prospective surveillance project for investigating the antimicrobial resistance and molecular epidemiology of major MDR bacterial pathogens causing nosocomial infections between August 2011 and July 2012. Twenty-seven hospitals from 26 provinces in mainland China participated in the surveillance voluntarily (see Acknowledgments section for full hospital list) (Fig. 1). Non-duplicate MDR-PA isolates causing nosocomial infections (from either hospitalized patients (≥48 h after admission) or in patients with a history of more than 48-h hospitalization within the last 30 days)^31^ were consecutively collected in the study (Fig. 1), and isolates from sputum and from screening swabs (for example throat swab and rectal swab) were excluded. For each MDR-PA isolate, clinical data was obtained from medical records by the collecting hospital, and completed on a standard electronic report form. All the isolates meeting the inclusion criteria were forwarded to our central laboratory (Department of Clinical Laboratory, Peking Union Medical College Hospital) for confirmative identification, further antibiotic susceptibility testing, carbapenemase screening and genotyping. Isolates were stored at −80 °C prior to testing.

### Antimicrobial susceptibility testing

At each participating hospital, antimicrobial susceptibility testing was performed according to local routine workflows. At the central laboratory, the minimum inhibitory concentrations (MICs) of nine antimicrobial agents against MDR-PA were determined for each isolate by broth micro-dilution method according to the Clinical and Laboratory Standards Institute (CLSI) guidelines [M7-A10]. The drugs tested included: aminoglycosides (amikacin), antipseudomonal cephalosporins (ceftazidime and cefepime), carbapenems (imipenem and meropenem), fluoroquinolones (ciprofloxacin), antipseudomonal β-lactams/β-lactamase inhibitors (piperacillin/tazobactam and cefoperazone/sulbactam), monobactams (aztreonam) and polymyxins (colistin). *Escherichia coli* ATCC 25922 and *P. aeruginosa* ATCC 27853 were used as quality controls at each run and their MICs were within recommended range. Interpretative criteria were consistent with CLSI document M100-S25.

The central laboratory classified MDR and XDR resistance patterns in *P. aeruginosa* according to proposed interim definitions^32^. For the eight antimicrobial categories selected for *P. aeruginosa* MDR and XDR definition, the isolates were defined as MDR when non-susceptible to ≥1 agent in ≥3 antimicrobial categories; and the isolates were defined as XDR when non-susceptible to ≥1 agent in all but ≤2 antimicrobial categories (i.e. bacterial isolates remain susceptible to only one or two categories)^32^.

### MLST and PFGE typing

DNA extraction and MLST was performed according to the protocol published by Curran *et al.*^33^. Standard DNA amplification of the seven housekeeping genes (*acsA*, *aroE*, *guaA*, *mutL*, *nuoD*, *ppsA*, and *trpE*) was performed for all isolates. The PCR products were sequenced in both directions using the DNA analyser ABI 3730XL system (Applied Biosystems, Foster City, CA). The nucleotide sequences were compared to existing sequences in the MLST database (www.pubmlst.org/paeruginosa) for assignment of allelic numbers. The isolates were assigned a ST number according to their allelic profiles. The phylogenetic analysis was performed by the MLST clustering software eBURST v3.0 (http://eburst.mlst.net/). Novel alleles in each novel ST were confirmed twice by sequencing in both directions and then submitted to MLST database.

To further investigate the genetic relationship of the isolates belonging to the most common ST, and to have some insight into intra-hospital epidemiology of isolates, PFGE analysis was performed as described by Hu *et al.*^34^. Briefly, genomic DNA was digested with 10 U restriction enzyme *Spe*I (New England BioLabs Inc.) and fragments separated by using CHEF MAPPER system (Bio-Rad, Richmond, USA). Dendrogram and cluster analysis were performed by BioNumerics software. Percentage similarities were identified on a dendrogram derived with the un-weighted pair-group method with arithmetic means (UPGMA) and Dice coefficients. The PFGE patterns of isolates with coefficient of similarity ≥90% were considered to belong to the same genotype^35^.

### Screening of carbapenemase producing isolates

The presence of acquired carbapenemases was determined by genetic approaches for the 233 carbapenem-resistant isolates. Described specific primers and conditions were used to amplify *bla*_VIM_, *bla*_IPM_, *bla*_KPC_, *bla*_NDM_, *bla*_NMC_, *bla*_SME_, *bla*_IMI_, *bla*_GES_, *bla*_SPM_, *bla*_GIM_*, bla*_SIM_, *bla*_OXA-24,_ or closely related carbapenemase genes^36^,^37^. The positive amplification products were sequenced and the results compared with those available in the GenBank database (www.ncbi.nil.gov/BLAST). Multiple-sequence alignments were performed with the CLC Sequence Viewer (version 7.0.2).

## Additional Information

**How to cite this article**: Fan, X. *et al.* Diverse Genetic Background of Multidrug-Resistant *Pseudomonas aeruginosa* from Mainland China, and Emergence of an Extensively Drug-Resistant ST292 Clone in Kunming. *Sci. Rep.* **6**, 26522; doi: 10.1038/srep26522 (2016).

## Supplementary Material

Supplementary Information

## Figures and Tables

**Table 1:** Specimen sources of multidrug resistant *Pseudomonas aeruginosa* and carbapenemases producers collected in mainland China.

Specimen source	No. of isolates	No. of carbapenemases producer (%)	Carbapenemase type (no.)
Pus (from abscesses)	49	4 (8.2%)	IMP-9 (2); VIM-2 (2)
Blood	44	3 (6.8%)	IMP-1 (2); VIM-2 (1)
Urine	42	8 (19.0%)	IMP-9 (5);VIM-2 (2); KPC-2 (1)
BALF	32	2 (6.3%)	IMP-9 (2)
Venous catheter	19	1 (5.3%)	IMP-9 (1)
Ascitic fluid	15	0	None
Surgery incision	15	0	None
Bile	14	0	None
Hydrothorax	12	1 (8.3%)	IMP-10 (1)
Cerebrospinal fluid	8	0	None
Tissue	2	0	None
Peritoneal dialysate fluid	1	0	None
Bone marrow	1	0	None
Total	254	19 (7.5%)	IMP-9 (10); VIM-2 (5); IMP-1 (2); KPC-2 (1); IMP10 (1)

Abbreviation: BALF, bronchoalveolar lavage fluid.

**Table 2:** Results of *in vitro* antimicrobial susceptibility tests of multidrug resistant *Pseudomonas aeruginosa* isolates from mainland China.

Antimicrobial agent	S%	I%	R%	MIC_50_ (mg/L)
Cefoperazone/Sulbactam	N/A	N/A	N/A	>32
Piperacillin/Tazobactam	6.7	20.5	72.8	128
Ceftazidime	20.9	16.1	63.0	32
Cefepime	6.7	18.1	75.2	>32
Aztreonam	10.6	13.8	75.6	>16
Imipenem	13.8	5.9	80.3	>8
Meropenem	15.0	9.0	76.0	>8
Amikacin	65.0	1.5	33.5	8
Ciprofloxacin	19.7	7.1	73.2	16
Colistin	72.4	19.7	7.9	2

Abbreviations: N/A, not applicable; S%, antimicrobial agents susceptible rate; I%, antimicrobial agents intermediate rate; R%, antimicrobial agents resistant rate.

**Table 3:** Information on carbapenemase producers detected in this study.

Carbapenemase (no. of isolates)	ST	Gender	Age	Hospitals^a^	Specimen source	Ward	Province	Resistant profile	Collection date
IMP-9 (10)	357	male	55	HN	Urine	Neurosurgery ward	Hainan	PCFAIMKR	2012/2/17
	357	male	27	HN	Urine	Neurosurgery ward	Hainan	PCFAIMKR	2012/4/2
	357	female	58	HN	Urine	Neurosurgery ward	Hainan	PCFAIMKR	2012/3/12
	919	female	27	SZ	BALF	ICU	Guangdong	PCF IMKR	2012/5/14
	919	male	84	SZ	BALF	ICU	Guangdong	PCF IMKR	2012/6/26
	267	male	49	TJ	Urine	Physical and Rehabilitation ward	Hubei	PCFAIMKR	2011/10/21
	273	female	62	ZZ	Urine	Physical and Rehabilitation ward	Henan	PCF IMK	2012/3/23
	773	male	25	XY	Pus	ICU	Hunan	CFA MK	2012/9/25
	1967	male	61	SC	Pus	Burn ward	Sichuan	PCF MKR	2011/9/8
	1973	male	12	XY	Venous catheter	Pediatrics ward	Hunan	PCF IMK	2011/10/24
IMP-1 (2)	463	male	17	SX	Blood	ICU	Shanxi	PCFAIM R	2012/5/15
	1420	male	39	ZR	Blood	Burn ward	Beijing	PCFAIMKR	2011/2/16
IMP10 (1)	1976	male	36	ZZ	Hydrothorax	Thoracic surgery ward	Henan	CFAIM R	2012/6/14
VIM2 (5)	390	male	0	XY	Pus	Neonatology ward	Hunan	PC IM	2012/6/12
	882	male	48	XY	Urine	ICU	Hunan	PCF IMK L	2012/7/22
	1974	male	45	XY	Blood	ICU	Hunan	PCFAIM	2012/8/13
	277	female	66	SC	Pus	Physical and Rehabilitation ward	Sichuan	P IM R	2011/9/24
	234	female	81	SD	Urine	ICU	Shandong	PCF IM	2011/10/21
KPC (1)	463	male	68	Z1	Urine	Gastrointestinal Surgery ward	Zhejiang	PCFAIM R	2012/6/2

Abbreviations: P, Piperacillin/Tazobactam; C, Ceftazidime; F, Cefepime; A, Aztreonam; I, Imipenem; M, Meropenem; K, Amikacin R, Ciprofloxacin; L, Colistin.

^a^The two letters are the abbreviation of the hospital that participated in this program. The full hospital name can be found in the Acknowledgements section.
