# Supplementary material for: Diverse Genetic Background of Multidrug-Resistant Pseudomonas aeruginosa from Mainland China, and Emergence of an Extensively Drug-Resistant ST292 Clone in Kunming
Source: Sci Rep. 2016 May 20;6:26522. doi: 10.1038/srep26522 (PMC4873832; doi:10.1038/srep26522)
Supplement: Supplementary Information [file srep26522-s1.pdf]

Diverse Genetic Background of Multidrug-Resistant *Pseudomonas aeruginosa* from Mainland China, and Emergence of an Extensively Drug-Resistant ST292 Clone in Kunming

Xin Fan[1,2], Yue Wu[1,2], Meng Xiao[1], Zhi-Peng Xu[1], Timothy Kudinha[3,4], Alda Bazaj[1], Fanrong Kong[4], and Ying-Chun Xu[1,*]

[1]Department of Clinical Laboratory, Peking Union Medical College Hospital, Chinese Academy of Medical Sciences, Beijing, China;

[2]Graduate School, Peking Union Medical College, Chinese Academy of Medical Sciences, Beijing, China;

[3]Charles Sturt University, Leeds Parade, Orange, New South Wales, Australia;

[4]Centre for Infectious Diseases and Microbiology Laboratory Services, ICPMR – Pathology West, University of Sydney, Westmead Hospital, Darcy Road, Westmead, New South Wales, NSW 2145, Australia

Supplementary Table S1. New sequence types (STs) and allelic profiles found in this study.

Isolate ID no.	ST	*acsA*	*aroE*	*guaA*	*mutL*	*nuoD*	*ppsA*	*trpE*	Gender[a]	Age	Collection year	Collection month	Comments
GPMDP018	1950	40	22	11	14	4	4	7	M	65	2012	April	
HBMDP006	1956	1	4	1	11	4	10	10	F	63	2011	November	
HBMDP020	1960	16	5	77	3	1	4	7	M	88	2012	April	
HNMDP002	1963	18	5	5	3	1	17	13	F	71	2011	July	
HNMDP015	1964	16	66	11	3	4	29	7	M	40	2011	October	
HZMDP011	1965	158	4	1	10	3	6	3	M	77	2011	September	
SCMDP001	1966	6	5	11	3	4	3	141	M	87	2011	August	
SCMDP004	1967	39	166	41	5	4	6	7	M	61	2011	September	IMP-9-producer
SDMDP037	1968	17	166	6	157	4	4	13	M	74	2012	June	
SJMDP015	1969	111	30	31	141	30	24	55	M	57	2012	March	
SZMDP006	1970	11	10	36	3	4	38	3	M	90	2011	November	
TJMDP022	1971	32	190	3	62	8	7	26	M	63	2012	March	
XYMDP001	1972	17	134	1	3	4	15	7	F	61	2012	June	

XYMDP012	1973	1	5	91	7	4	12	7	M	12	2011	October	IMP-9-producer
XYMDP029	1974	39	5	6	11	4	7	193	M	45	2012	August	VIM-2-producer
ZSMDP007	1975	25	76	36	11	44	4	10	F	53	2011	October	
ZZMDP021	1976	17	76	11	72	3	10	3	M	36	2012	June	IMP-10-producer

[a] M, male; F, female.
